# Accuracy and precision of ultrasound shear wave elasticity measurements according to target elasticity and acquisition depth: A phantom study

**DOI:** 10.1371/journal.pone.0219621

**Published:** 2019-07-11

**Authors:** Chong Hyun Suh, Hee Mang Yoon, Seung Chai Jung, Young Jun Choi

**Affiliations:** Department of Radiology and Research Institute of Radiology, University of Ulsan College of Medicine, Asan Medical Center, Asanbyeongwon-Gil, Songpa-Gu, Seoul, Republic of Korea; University of Montreal, CANADA

## Abstract

**Objective:**

To investigate the accuracy and precision of ultrasound shear wave elasticity measurements as a function of target elasticity and acquisition depth.

**Materials and methods:**

Using five ultrasound systems (VTQ, VTIQ, EPIQ 5, Aixplorer, and Aplio 500), two operators independently measured shear wave elasticities in two phantoms containing five different target elasticities (8±3, 14±4, 25±6, 45±8, and 80±12 kPa) at depths of 15, 30, 35, and 60 mm. Accuracy was assessed by evaluating measurement errors and the proportions of outliers, while factors affecting accuracy were assessed using logistic regression analysis. Measurement errors were defined as differences between the measured values and 1) the margins of the target elasticity, and 2) the median values of the target elasticity. Outliers were defined as measured values outside the margins of the target elasticity. Precision was assessed by calculating the reproducibility of measurements using the within-subject coefficient of variation (wCV).

**Results:**

Mean measurement errors and the proportions of outliers were higher for high than for low target elasticities (*p*<0.001), but did not differ in relation to acquisition depth, either within an elastography system or across the different systems. Logistic regression analysis showed that target elasticity (*p*<0.001) significantly affected accuracy, whereas acquisition depth (*p*>0.05) did not. The wCV for the 80±12 kPa target (31.33%) was significantly higher than that for lower elasticity targets (6.96–10.43 kPa; *p*<0.001). The wCV did not differ across acquisition depths. The individual elastography systems showed consistent results.

**Conclusions:**

Targets with high elasticity showed lower accuracy and lower precision than targets with low elasticity, while acquisition depth did not show consistent patterns in either accuracy or precision.

## Introduction

Ultrasound (US) elastography is a noninvasive imaging modality for assessment of tissue stiffness. US elastography techniques can be classified as shear wave or strain imaging [1,2], and three modalities are available for shear wave imaging: transient elastography, point shear wave speed measurement, and shear wave speed imaging. Shear waves are generated by controlled external vibrations in transient elastography (Fibroscan, Echogen), and by acoustic radiation force impulses in point shear wave speed measurement (Virtual Touch Quantification [VTQ]; EPIQ 5) and shear wave speed imaging (Virtual Touch Image Quantification [VTIQ]; Aixplorer; Aplio) [1].

New US elastography systems are currently being developed, and US shear wave elastography has been approved for clinical use by the United States Food and Drug Administration (FDA). To validate shear wave elastography as a quantitative imaging biomarker, its accuracy and precision should be guaranteed. Accuracy and precision are essential and fundamental for quantitative imaging modalities [3].

As shear wave elastography is currently being utilized in clinical practice [4–10], determination of its accuracy and precision is crucial for measurements of tissue stiffness, especially those of the liver, thyroid, breast, and prostate [4–10]. A previous study assessing precision reported higher variability for the higher elasticity phantom [11]. Additionally, tissue attenuation may dampen ultrasound signals as a function of acquisition depth, limiting the accurate measurement of deeper tissue or organs [12]. However, to our knowledge, no phantom studies have evaluated the influence of target elasticity and acquisition depth on the accuracy of shear wave elastography. This study therefore investigated the effects of target elasticity and acquisition depth on the accuracy and precision of US shear wave elasticity measurements.

## Materials and methods

### Phantoms

Model 049 and 049A QA phantoms were obtained from Computerized Imaging Reference Systems (CIRS; Norfolk, Virginia, USA). These phantoms are manufactured using Zerdine, a solid-elastic polymer with elasticity properties that can be controlled independently of its acoustic properties [13]. The model 049 QA phantom contains sets of spherical mapping targets of 10 mm (depth, 15 mm) and 20 mm diameter (depth, 35 mm). The model 049A QA phantom contains sets of stepped cylinders that vary in diameter from 1.6 to 16.7 mm. The targets of 6.5 mm, 10.4 mm, and 16.7 mm diameters were used in this study. The stepped cylinders in each set are located at depths from 30 to 60 mm, with these depths referring to the centers of the spherical and cylindrical targets. Both phantoms contain materials of five different elasticities, consisting of four types of simulated lesions with elasticities of 8 ± 3, 14 ± 4, 45 ± 8, and 80 ± 12 kPa, and background material of elasticity 25 ± 6 kPa. This study performed measurements on five targets, of elasticities 8 ± 3, 14 ± 4, 25 ± 6, 45 ± 8 kPa, and 80 ± 12 kPa.

### Shear wave elasticity measurements

**Fig 1:**
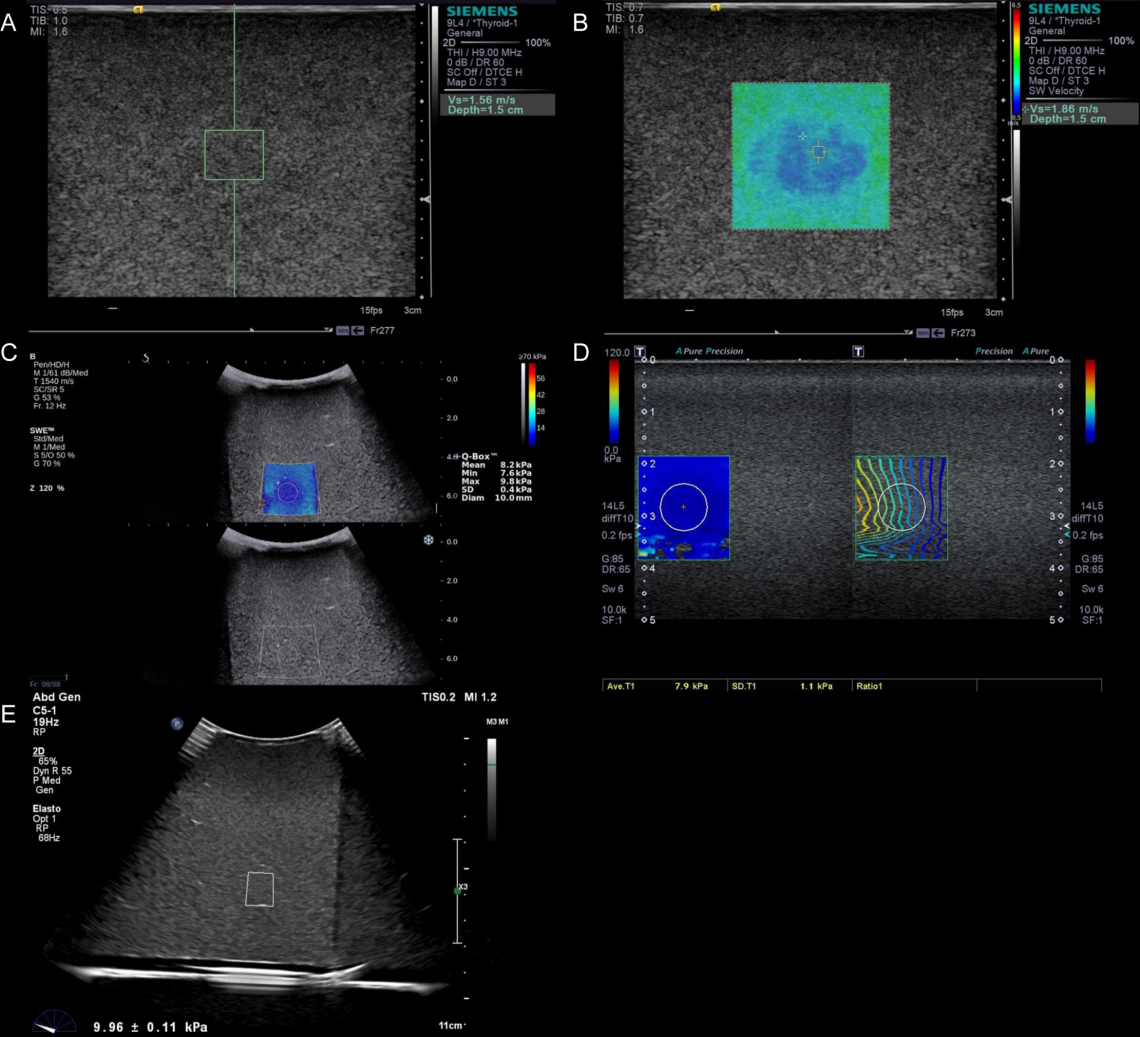
Measurements of shear wave elasticities in a target of 8 ± 3 kPa using four different machines. (A and B) A linear transducer and the VTQ system and a linear transducer and the VTIQ system (ACUSON S2000, Siemens Healthcare, Erlangen, Germany) at depths of 15 mm, (C) a curved transducer and the Aixplorer system (Supersonic Imagine, Aix Provence, France) at a depth of 60 mm, (D) a linear transducer and the Aplio 500 system (Toshiba Medical Systems, Tochigi-ken, Japan) at a depth of 30 mm, and (E) a curved transducer and the EPIQ 5 system (Philips Medical System, Best, the Netherlands) at a depth of 60 mm.

Shear wave imaging was performed at a single institution using recently introduced shear wave elastography systems including the ACUSON S2000 (VTQ and VTIQ, Siemens Healthcare, Erlangen, Germany), EPIQ 5 (Philips Medical System, Best, the Netherlands), Aixplorer (Supersonic Imagine, Aix Provence, France), and Aplio 500 (Toshiba Medical Systems, Tochigi-ken, Japan; Fig 1). Both linear and curved transducers were available for use with the Aixplorer (SC6-1 curved transducer, SL10-2 linear transducer) and Aplio 500 (375BT curved transducer, 1005BT linear transducer); however, only a linear transducer (9L4 linear transducer) was available for the ACUSON S2000, and only a curved transducer (C5-1 curved transducer) was available for the EPIQ 5.

Measurements were performed by two operators, with operator 1 being a neuroradiologist with 1 year of experience with US elastography, and operator 2 being a pediatric radiologist with 5 years of experience with US elastography. Each operator acquired a series of ten consecutive shear wave elasticity measurements on each of the systems, and the mean of these ten measurements on each system, each depth, and each transducer was calculated. A total of 14 series of ten measurements were performed by each observer for one target. Among eight series of ten measurements with a linear transducer, four series of ten measurements were performed at 15 mm and 35 mm, respectively. Among six series of ten measurements with a curved transducer, three series of ten measurements were performed at 30 mm and 60 mm, respectively.

Shear wave elasticities were measured on the EPIQ 5, Aixplorer, and Aplio 500 systems as kPa, and as shear wave velocities (m/s) on the ACUSON S2000 system, with shear wave velocities (m/s) converted to kPa using the formula *E = 3pc*^*2*^, where *E* is stiffness (i.e., Young’s elastic modulus [kPa]), *c* is the shear wave velocity (m/s), and *p* is the density (kg/m^3^) [2,14–16]. The density of most soft tissues is approximately equal to that of water (1000 kg/m^3^) [2,14–16].

For the 049 phantom, shear wave elasticity was measured on five different elasticity targets (8 ± 3, 14 ± 4, 25 ± 6, 45 ± 8 kPa, and 80 ± 12 kPa) at two depths of 15 (target size: 10 mm) and 35 mm (target size: 20 mm) using a linear transducer. For the 049A phantom, shear wave velocity was measured on the same five elasticity targets with target size of 16.7 mm at depths of 30 and 60 mm using a curved transducer. Regions of interest (ROIs) were placed onto homogeneous target regions of the phantom. For the Aixplorer and Aplio 500 systems, ROIs with respective maximum diameters of 10 and 9 mm were used, whereas, for the VTQ, VTIQ, and EPIQ 5 systems, the ROI sizes were fixed (5 × 6, 1.5 × 1.5, and 10 × 12 mm, respectively). ROIs were placed onto B-mode images, in the center of the targets at the predetermined acquisition depths. For the phantom background (25 kPa), elasticity was measured in the same scanned region using all techniques. In the shear wave speed imaging (VTIQ, Aixplorer, and Aplio 500), images were fully filled, in color, for all measurements. Additionally, we measured small (10.4 mm and 6.5 mm) targets in the 049A phantom using curved Aixplorer and Aplio 500 probes, which can decrease the size of the ROIs. We used an ROI diameter of 8 mm for the target size of 10.4 mm, and an ROI diameter of 5 mm for the target size of 6.5 mm.

### Statistical analysis

The shear wave elasticity of each phantom was measured ten consecutive times by each operator for each imaging depth and each elasticity target, with the results being summarized as the mean value and standard deviation.

To assess the accuracy of measurements, measurement errors and the proportions of outliers were calculated and compared among target elasticities and acquisition depths. Measurement errors were defined as the differences between the measured values and the margins of the target elasticity values, and outliers were defined as measured values outside of the margins of the target elasticity values. Results were compared by repeated measures ANOVA and McNemar’s test. Factors potentially affecting accuracy were assessed by logistic regression analysis according to the target elasticities and acquisition depths.

To assess precision, repeatability was calculated by determining the within-subject coefficient of variation (wCV). This coefficient is indicative of the within-subject variability of parameters and is expressed as a percentage; it was obtained by dividing the within-subject standard deviation by the group mean. A wCV > 50% was regarded as indicating unreliability for clinical implementation [17]. The wCVs were obtained according to the target elasticities and acquisition depths, and were compared among them. The equality of the wCVs was assessed using an asymptotic test [18]. In addition, to investigate the degree of variation across multiple measurements of a single target with an individual elastography system, the coefficients of variation for each measurement were calculated using the following equation: coefficient of variation = standard deviation / mean value × 100% [14]. As the coefficients of variation became larger, the reliability of any single measurement decreased.

Accuracy and precision according to linear and curved transducers was evaluated. In addition, accuracy and precision for small targets (10.4 mm and 6.5 mm) was also evaluated. Interobserver agreement was evaluated by Bland-Altman analysis, and the differences between the measurements of different observers are reported as mean differences and the 95% limits of agreement in elasticity for each shear wave imaging technique and transducer [19]. All statistical analyses were performed using R version 3.4.1 (The R Foundation for Statistical Computing) with the “EntropyExplorer” package [20], and MedCalc software (version 18.6). A *p* value < 0.05 was regarded as statistically significant.

## Results

### Accuracy

The measurement errors derived from differences between the measured values and the margins of the target elasticity values of 8 ± 3, 14 ± 4, and 25 ± 6 kPa were all less than 1 kPa, being 0.10 kPa (95% CI, 0.06–0.15 kPa), 0.19 kPa (95% CI, 0.14–0.23 kPa), and 0.51 kPa (95% CI, 0.41–0.61 kPa), respectively (Table 1). The measurement error for the 45 ± 8 kPa target was 5.99 kPa (95% CI, 5.44–6.55 kPa), which was significantly higher than that for the targets of 8 ± 3, 14 ± 4, and 25 ± 6 kPa (*p* < 0.001). The measurement error for the 80 ± 12 kPa target was 21.01 kPa (95% CI, 18.59–23.43 kPa), which was also significantly higher than that for the targets of 8 ± 3, 14 ± 4, and 25 ± 6 kPa (*p* < 0.001). In addition, the measurement error for the 45 ± 8 kPa and 80 ± 12 kPa targets were consistently lower than the nominal value from the manufacturer of the phantom. In post-hoc analyses, higher target elasticities showed significantly higher measurement errors (*p* < 0.001, except for *p* = 0.014 between 8 ± 3 and 14 ± 4 kPa). Results from the individual elastography systems showed that the measurement error for the elasticity targets of 45 ± 8 and 80 ± 12 kPa were consistently higher than that for targets of 8 ± 3, 14 ± 4, and 25 ± 6 kPa, with this being the case for all five elastography systems (*p* < 0.0083; Table 2).

**Table 1:** Measurement errors derived from differences between measured values and the margins of the target elasticity values, and proportions of outliers according to target elasticity and acquisition depth.

	Measurement errors (kPa)	*P* value	Proportions of outliers (%)	*P* value
**Target elasticity^†^**				
**8 ± 3 kPa**	0.10		3.6 (1 of 28)	
**14 ± 4 kPa**	0.19		32.1 (9 of 28)	
**25 ± 6 kPa**	0.51		39.3 (11 of 28)	
**45 ± 8 kPa**	5.99	< 0.001	82.1 (23 of 28)	< 0.001
**80 ± 12 kPa**	21.01	< 0.001	82.1 (23 of 28)	< 0.001
**Acquisition depth**^††^				
8 ± 3 kPa				
**15 mm**	0.04		0 (0 of 8)	
**30 mm**	0.27	0.002	16.7 (1 of 6)	0.248
**35 mm**	0.06	1.000	0 (0 of 8)	NA
**60 mm**	0.07	1.000	0 (0 of 6)	NA
14 ± 4 kPa				
**15 mm**	0.00		0 (0 of 8)	
**30 mm**	0.20	< 0.001	50 (3 of 6)	0.030
**35 mm**	0.43	< 0.001	62.5 (5 of 8)	0.009
**60 mm**	0.08	0.770	16.7 (1 of 6)	0.248
25 ± 6 kPa				
**15 mm**	0.41		62.5 (5 of 8)	
**30 mm**	0.02	0.024	0 (0 of 6)	0.020
**35 mm**	1.06	< 0.001	50 (4 of 8)	0.626
**60 mm**	0.39	1.000	16.7 (1 of 6)	0.099
45 ± 8 kPa				
**15 mm**	9.06		75 (6 of 8)	
**30 mm**	4.50	< 0.001	83.3 (5 of 6)	0.718
**35 mm**	4.22	< 0.001	87.5 (7 of 8)	0.535
**60 mm**	5.76	< 0.001	83.3 (5 of 6)	0.718
**80 ± 12 kPa**				
**15 mm**	24.78		87.5 (7 of 8)	
**30 mm**	18.32	0.398	66.7 (4 of 6)	0.366
**35 mm**	18.53	0.330	75 (6 of 8)	0.535
**60 mm**	21.98	1.000	100 (6 of 6)	0.387

^†^ Mean measurement errors and proportions of outliers were higher for targets with high (45 ± 8 and 80 ± 12 kPa) rather than low elasticities (8 ± 3, 14 ± 4, and 25 ± 6 kPa) (*p* < 0.001).

^††^ Mean measurement errors and proportions of outliers did not differ across acquisition depths.

**Table 2:** Measurement errors derived from differences between measured values and the margins of the target elasticity values, and proportions of outliers according to target elasticity and elastography system.

	**Measurement errors (kPa)**^†^	***P* value**	**Proportions of outliers (%)**^††^	***P* value**
**VTQ**				
**8 ± 3 kPa**	0	< 0.0083	0 (0 of 4)	
**14 ± 4 kPa**	0.56	< 0.0083	50 (2 of 4)	
**25 ± 6 kPa**	1.26	< 0.0083	100 (4 of 4)	
**45 ± 8 kPa**	10.50		100 (4 of 4)	0.0082
**80 ± 12 kPa**	55.25		100 (4 of 4)	
**VTIQ**				
**8 ± 3 kPa**	0.19	< 0.0083	0 (0 of 4)	
**14 ± 4 kPa**	0	< 0.0083	0 (0 of 4)	
**25 ± 6 kPa**	0.07	< 0.0083	0 (0 of 4)	
**45 ± 8 kPa**	0.99		50 (2 of 4)	0.1266
**80 ± 12 kPa**	10.81		75 (3 of 4)	
**EPIQ 5**				
**8 ± 3 kPa**	0.52	< 0.0083	25 (1 of 4)	
**14 ± 4 kPa**	0.02	< 0.0083	0 (0 of 4)	
**25 ± 6 kPa**	0.02	< 0.0083	0 (0 of 4)	
**45 ± 8 kPa**	7.79		100 (4 of 4)	0.0404
**80 ± 12 kPa**	31.99		100 (4 of 4)	
**Aixplorer**				
**8 ± 3 kPa**	0	< 0.0083	0 (0 of 8)	
**14 ± 4 kPa**	0.22	< 0.0083	50 (4 of 8)	
**25 ± 6 kPa**	0.38	< 0.0083	25 (4 of 8)	
**45 ± 8 kPa**	3.46		100 (8 of 8)	0.0001
**80 ± 12 kPa**	18.05		100 (8 of 8)	
**Aplio 500**				
**8 ± 3 kPa**	0	< 0.0083	0 (0 of 8)	
**14 ± 4 kPa**	0.17	< 0.0083	38 (3 of 8)	
**25 ± 6 kPa**	0.87	< 0.0083	63 (5 of 8)	
**45 ± 8 kPa**	5.04		63 (5 of 8)	0.0090
**80 ± 12** kPa	6.45		50 (4 of 8)	

^†^ In post-hoc analyses, the measurement error for the target with an elasticity of 45 ± 8 and 80 ± 12 kPa were consistently higher than that for targets of 8 ± 3, 14 ± 4, and 25 ± 6 kPa (*p* < 0.0083), with this being the case for all elastography systems.

^††^ Proportions of outliers did not differ across target elasticities.

The measurement errors derived from the differences between measured values and the median target elasticity values of 8 ± 3, 14 ± 4, and 25 ± 6 kPa were less than or equal to 5 kPa, being 1.31 kPa (95% CI, 1.17–1.46 kPa), 2.96 kPa (95% CI, 2.80–3.12 kPa), and 5.00 kPa (95% CI, 4.71–5.28 kPa), respectively, while that for the elasticity targets of 45 ± 8 and 80 ± 12 kPa were 13.65 kPa (95% CI, 12.90–14.40 kPa) and 31.79 kPa (95% CI, 29.20–34.37 kPa; Table 3). In post-hoc analyses, higher target elasticities showed significantly higher measurement errors (*p* < 0.001). Results from the individual elastography systems also showed that the measurement errors derived from the difference between the measured values and the median target elasticity value were consistently higher for the elasticity target of 45 ± 8 kPa and 80 ± 12 kPa than for the targets of 8 ± 3, 14 ± 4, and 25 ± 6 kPa, with this being the case for all five elastography systems (*p* < 0.0083; Table 4).

**Table 3:** Measurement errors derived from differences between measured values and median target elasticity values according to target elasticity and acquisition depth.

	Measurement errors (kPa)	*P* value
**Target elasticity^†^**		
**8 ± 3 kPa**	1.31	
**14 ± 4 kPa**	2.96	
**25 ± 6 kPa**	5.00	
**45 ± 8 kPa**	13.65	< 0.001
**80** ± **12** kPa	31.79	< 0.001
**Acquisition depth**^††^		
8 ± 3 kPa		
**15 mm**	0.78	
**30 mm**	1.75	0.001
**35 mm**	0.80	1.000
**60 mm**	1.62	< 0.001
14 ± 4 kPa		
**15 mm**	2.27	
**30 mm**	3.46	< 0.001
**35 mm**	3.66	< 0.001
**60 mm**	2.86	0.004
25 ± 6 kPa		
**15 mm**	5.90	
**30 mm**	3.43	< 0.001
**35 mm**	6.24	1.000
**60 mm**	4.75	0.0239
45 ± 8 kPa		
**15 mm**	17.66	
**30 mm**	12.21	< 0.001
**35 mm**	11.25	< 0.001
**60 mm**	13.25	< 0.001
**80 ± 12 kPa**		
**15 mm**	36.14	
**30 mm**	27.22	0.104
**35 mm**	29.53	0.339
**60 mm**	33.54	1.000

^†^ Mean measurement errors were higher for targets with high (45 ± 8 kPa and 80 ± 12 kPa) rather than low elasticities (8 ± 3, 14 ± 4, and 25 ± 6 kPa) (*p* < 0.001).

^††^ Mean measurement errors did not differ across acquisition depths.

**Table 4:** Measurement errors derived from differences between measured values and median target elasticity values according to target elasticity and different elastography systems.

	Measurement errors (kPa)^†^	*P* value
**VTQ**		
**8 ± 3 kPa**	0.66	< 0.0083
**14 ± 4 kPa**	3.73	< 0.0083
**25 ± 6 kPa**	7.25	< 0.0083
**45 ± 8 kPa**	18.50	
**80 ± 12 kPa**	67.25	
**VTIQ**		
**8 ± 3 kPa**	2.73	< 0.0083
**14 ± 4 kPa**	1.35	< 0.0083
**25 ± 6 kPa**	2.27	< 0.0083
**45 ± 8 kPa**	7.94	
**80 ± 12 kPa**	21.55	
**EPIQ 5**		
**8 ± 3 kPa**	2.93	< 0.0083
**14 ± 4 kPa**	1.91	< 0.0083
**25 ± 6 kPa**	2.42	< 0.0083
**45 ± 8 kPa**	15.64	
**80 ± 12 kPa**	43.02	
**Aixplorer**		
**8 ± 3 kPa**	0.95	< 0.0083
**14 ± 4 kPa**	3.44	< 0.0083
**25 ± 6 kPa**	5.28	< 0.0083
**45 ± 8 kPa**	14.29	
**80 ± 12 kPa**	30.05	
**Aplio 500**		
**8 ± 3 kPa**	0.49	< 0.0083
**14 ± 4 kPa**	3.42	< 0.0083
**25 ± 6 kPa**	6.24	< 0.0083
**45 ± 8 kPa**	12.17	
**80 ± 12 kPa**	15.29	

^†^ In post-hoc analyses, the measurement error for the target with an elasticity of 45 ± 8 and 80 ± 12 kPa were consistently higher than those for targets of 8 ± 3, 14 ± 4, and 25 ± 6 kPa (*p* < 0.0083), with this being the case for all elastography systems.

The overall proportion of outliers was 47.9% (67 of 140; Table 1). The 45 ± 8 kPa and 80 ± 12 kPa elasticity target (82.1%, 23 of 28, each) showed significantly higher proportions of outliers than the targets with elasticities of 8 ± 3 kPa (3.6%, 1 of 28), 14 ± 4 kPa (32.1%, 9 of 28), and 25 ± 6 kPa (39.3%, 11 of 28; *p* < 0.001). According to the individual elastography systems, the proportions of outliers did not differ across target elasticities (Table 2). Measurement errors and the proportions of outliers were found to be independent of acquisition depth (Table 1); accuracy was not compromised by increased acquisition depth.

Logistic regression analysis was performed to determine the effects of target elasticity and acquisition depth on accuracy (Table 5); this showed that target elasticity significantly affected accuracy (*p* < 0.001), whereas acquisition depth did not (*p* > 0.05).

**Table 5:** Logistic regression analysis of ultrasound shear wave elasticity measurements according to target elasticity and acquisition depth.

Variable	Odds ratio^†^	95% confidence interval	*P* value
**Target elasticity**			
**8 ± 3 kPa**			
**14 ± 4 kPa**	4.65	2.89–7.48	< 0.001
**25 ± 6 kPa**	6.84	4.28–10.95	< 0.001
**45 ± 8 kPa**	44.43	26.79–73.70	< 0.001
**80 ± 12 kPa**	30.82	18.91–50.22	< 0.001
**Acquisition depth**			
**15 mm**			
**30 mm**	0.85	0.59–1.21	0.362
**35 mm**	1.19	0.85–1.76	1.187
**60 mm**	0.88	0.62–1.26	0.879

^†^ Compared with baseline target elasticity (8 ± 3 kPa) and acquisition depth (15 mm). Logistic regression analysis showed that target elasticity significantly affected accuracy (*p* < 0.001), whereas acquisition depth did not (*p* > 0.05).

### Precision

The wCVs and their 95% CIs for all measurements are summarized in S1 Table. The overall wCV for the reproducibility of all measurements was 31.26% (95% CI, 23.31*–*37.98). The wCV for the 80 ± 12 kPa target (31.33% [95% CI,–%) was significantly higher than that for the targets of 8 ± 3 kPa (6.96% [95% CI, 5.79–8.13%]), 14 ± 4 kPa (7.69% [95% CI, 6.15–9.38%], 25 ± 6 kPa (8.47% [95% CI, 7.02–10.01%], and 45 ± 8 kPa (10.43% [95% CI, 8.81–12.15%]; *p* < 0.001). S1 Fig and S2 Fig illustrate variations in shear wave elasticity measurements according to the different shear wave imaging techniques and transducers. In the elasticity targets of 8 ± 3, 14 ± 4, 25 ± 6, and 45 ± 8 kPa, the coefficients of variation were low, ranged from 0.4% to 2.7% for the VTQ (mean, 1.1% ± 0.6%), 0.6% to 6.2% for the Aixplorer (mean, 1.7% ± 2.0%), 1.3% to 5.9% for the VTIQ (mean, 2.9% ± 1.5%), 0.7% to 15.1% for the Aplio 500 (mean, 5.5% ± 4.0%), and 5.1% to 24.0% for the EPIQ 5 (mean, 11.1% ± 4.7%; S2 Table and S3 Table). However, in the 80 ± 12 kPa target, coefficients of variation were high, ranged from 7.5% to 58.9% for the VTQ, 1.8% to 62.1% for the Aixplorer, 2.6% to 72.6% for the VTIQ, 4.3% to 87.7% for the Aplio, and 14.5% to 143.4% for the EPIQ 5.

Results from the individual elastography systems showed that the coefficients of variation (S2 Table and S3 Table) did not significantly differ across different target elasticities. Furthermore, the wCVs did not significantly differ across acquisition depths (S1 Table); wCV was not compromised by increases in acquisition depth up to 60 mm.

### Accuracy and precision according to transducer

The overall measurement errors of linear and curved transducers were 5.86 kPa (95% CI, 4.97–6.74 kPa) and 5.16 kPa (95% CI, 4.20–6.11 kPa), respectively (S4 Table). There was no statistical difference between linear and curved transducers (*p* = 0.296). The measurement errors for the 45 ± 8 kPa and 80 ± 12 kPa targets were significantly higher than those for the targets of 8 ± 3, 14 ± 4, and 25 ± 6 kPa for both linear and curved transducers (*p* < 0.001). The wCV for the 8 ± 3, 14 ± 4, 25 ± 6, and 45 ± 8 kPa (5.22–12.00 kPa) were lower compared with those for the 80 ± 12 kPa target (16.43–38.93 kPa) for both linear and curved transducers (*p* < 0.001).

### Accuracy and precision for small targets

The low-elasticity targets of 8 ± 3 kPa, 14 ± 4 kPa, and 25 ± 6 kPa (0–2.69 kPa) were associated with a low degree of error; however, the high-elasticity targets of 45 ± 8 kPa and 80 ± 12 kPa (13.12–41.73 kPa; *p* < 0.016; S5 Table) were associated with a high degree of error. Also, the wCV for the 8 ± 3 kPa, 14 ± 4 kPa, 25 ± 6 kPa, and 45 ± 8 kPa targets (6.55–11.29 kPa) were lower compared with the 80 ± 12 kPa target (19.06–26.27 kPa) for small targets (*p* < 0.001).

### Interobserver agreement

Bland-Altman analysis of interobserver agreement showed a mean difference over all elasticity measurements of 4.2%, with a 95% limit of agreement of 4.2% ± 47.2%. Bland-Altman analysis showed slight mean differences in interobserver agreement for target elasticity of 8 ± 3, 14 ± 4, 25 ± 6, and 45 ± 8 kPa (range, 1.0–2.4%), whereas large mean difference for 80 ± 12 kPa target (17.1%). In addition, Bland-Altman analysis showed slight mean differences in interobserver agreement in relation to acquisition depth (range, 2.1–7.5%).

## Discussion

This study evaluated the accuracy and precision of US shear wave elasticity measurements for targets of different elasticities and at different acquisition depths. We found that targets with an elasticity of 45 ± 8 kPa and 80 ± 12 kPa showed a significantly higher proportion of outliers (82.1%, each) and higher measurement errors (5.99 and 21.01 kPa, respectively;) than targets with elasticities of 8 ± 3, 14 ± 4, and 25 ± 6 kPa. Logistic regression analysis showed that target elasticity significantly affected accuracy, whereas acquisition depth did not. The wCV for the 80 ± 12 kPa target (31.33%) was significantly higher than that for the targets of 8 ± 3, 14 ± 4, 25 ± 6, and 45 ± 8 kPa (6.96–10.43 kPa; *p* < 0.001). The wCVs did not significantly differ across acquisition depths, with individual elastography systems showing consistent results. Taken together, targets with high elasticity showed lower accuracy and lower precision than targets with low elasticity, while acquisition depth did not show consistent patterns in either accuracy or precision.

In this study, the target with the high elasticity targets (45 ± 8 kPa and 80 ± 12 kPa) yielded lower accuracy than the targets with lower elasticity. These high elasticity targets showed a significantly higher measurement error and higher proportion of measurement errors than targets of lower elasticity. Logistic regression analysis showed that target elasticity significantly affected accuracy. Moreover, the 80 ± 12 kPa target yielded lower precision than lower elasticity targets. Previous studies have reported high variability for high elasticity targets for shear wave elastography [11,21]. This phenomenon is probably due to the higher shear wave attenuation in high elasticity conditions [12]. Our results suggest the need for caution when measuring elasticity in lesions with high elasticity, which would include malignant lymph nodes. A previous meta-analysis reported that the cutoff values for differentiating malignant cervical lymph nodes from benign lymph nodes on shear wave elastography ranged from 19.4 to 57 kPa [5].

If we focus on target elasticities between 8 ± 3 and 25 ± 6 kPa, the current study results revealed measurement errors of only 0.10–0.51 kPa. In addition, data from the five individual elastography systems also demonstrated low measurement error (0.10–1.26 kPa). Moreover, our results demonstrated low wCVs (6.96–8.47%) for target elasticities between 8 ± 3 and 25 ± 6 kPa. These findings suggest the presence of high accuracy and reproducibility across five different elastography systems for target elasticities between 8 ± 3 and 25 ± 6 kPa, target elasticity values that are commonly encountered in daily clinical practice.

A previous study using the VTQ, VTIQ, and Aixplorer systems reported no significant trends between the coefficients of variation and acquisition depths of 10, 25, and 40 mm [22]. The present study also found that the wCV and coefficient of variation did not differ across acquisition depths. Despite increases in acquisition depth of up to 60 mm, the precision of the US elastography systems was not compromised. With regard to accuracy, the current study also revealed that measurement errors and the proportions of outliers were independent of acquisition depth, and the logistic regression analysis also showed that acquisition depth did not affect accuracy. Until now, no study had evaluated the accuracy of US shear wave elasticity measurements in relation to acquisition depth. First, a plausible explanation for our results could be that depth is not an explicit physical quantity, but it is the indirect effect of various confounding factors, such as US and shear wave attenuation, focusing acquisition depth, pulse energy, and other parameters. US shear waves degrade and distort through heterogeneous media with variable elasticities until they arrive at a target in vivo tissue, but US shear wave attenuation is less likely to occur with the uniform media of in vitro phantoms. In vivo heterogeneity may result in unpredictable and inconsistent acquisition depth results [23]. Therefore, phantom studies may be optimal for evaluating the performance of US elastography in a setting of the most important parameters such as elasticity, lesion size, and acquisition depths are known [21]. Second, the acquisition depths may not have been deep enough to reveal significant differences, unlike the elasticities. Although the acquisition depths could affect the shear wave measurements, the results using in vitro phantoms did not provide any consistent patterns in our and previous studies. In our study, we measured targets which have acquisition depth up to 60 mm and previous studies also did the same manner [11,22,24]. US elastography measurement is usually performed within the acquisition depth of 60 mm in the liver [4]. Therefore, our study results might have clinical implications for elasticity measurement.

This information may be clinically meaningful, especially in the absence of a particular target lesion, such as in liver parenchyma, where it may be hard to achieve the same acquisition depth between operators. Therefore, this study demonstrates that US elastography has high reproducibility and accuracy, regardless of the acquisition depth, a finding that is important for daily clinical practice.

This study revealed that the wCVs of low elasticity targets (8 ± 3, 14 ± 4 25 ± 6 and 45 ± 8 kPa) were low (6.96–10.43%) and previous reports also showed low coefficients of variation (0% to 9%) [11,22,24]. In addition, interobserver agreement according to target elasticity and acquisition depth showed only very slight mean differences, a finding that was also in agreement with previous studies that showed high interobserver reliability for shear wave methods (intraclass correlation coefficients of 0.99–1.00) [11,24].

This study has several limitations. First, because this was a phantom study, we could not evaluate clinical conditions that could affect the results of the shear wave elasticity measurements. The US elastography phantoms did not have viscoelastic components like live soft tissues [23]. Without animal or human data, technical limitations may confound our conclusions. Our results must be verified by further studies using animal or human subjects. Second, as our hospital uses the ACUSON S2000 only for evaluation of the superficial neck, a curved transducer was not available, and a linear transducer had not been developed for the EPIQ 5 at the time of this study. Third, although we tried to evaluate various target elasticities and acquisition depths, only five different target elasticities and acquisition depths were evaluated, and these were only investigated using two phantoms. Therefore, it is necessary to use various phantoms with large sample size to evaluate elasticities and acquisition depths with both the US elastography systems used in this study and other ones. Fourth, we could not add an attenuating medium with a known attenuation coefficient on top of the phantoms. Further research using an attenuating medium will be needed.

## Conclusions

Targets with high elasticity showed lower accuracy and lower precision than targets with low elasticity, while acquisition depth did not show consistent patterns in either accuracy or precision.

## Supporting information

S1 Table Within-subject coefficients of variation (wCVs) for ultrasound shear wave elasticity measurements according to target elasticity and acquisition depth. (DOCX)

S2 Table Shear wave elasticity measurements for four different elasticity targets at two different depths, with measurements obtained using a linear transducer. (DOCX)

S3 Table Shear wave elasticity measurements for four different elasticity targets at two different depths, with measurements obtained using a curved transducer. (DOCX)

S4 Table Measurement errors derived from differences between measured values and the margins of the target elasticity values, proportions of outliers, and within-subject coefficients of variation (wCV) according to transducers. (DOCX)

S5 Table Measurement errors derived from differences between measured values and the margins of the target elasticity values, proportions of outliers, and within-subject coefficients of variation (wCV) according to target elasticity in small targets. (DOCX)

S1 Fig Variation in shear wave elasticity measurements according to different shear wave imaging techniques with linear transducers. A. a linear transducer of VTQ, B. a linear transducer of VTIQ, C. a linear transducer of Aixplorer, and D. a linear transducer of Aplio 500.
(TIF)

S2 Fig Variation in shear wave elasticity measurements according to different shear wave imaging techniques with curved transducers. A. a curved transducer of EPIQ 5, B. a curved transducer of Aixplorer, and C. a curved transducer of Aplio 500.
(TIF)
