# Supplementary material for: Accuracy and precision of ultrasound shear wave elasticity measurements according to target elasticity and acquisition depth: A phantom study
Source: PLoS One. 2019 Jul 11;14(7):e0219621. doi: 10.1371/journal.pone.0219621 (PMC6622533; doi:10.1371/journal.pone.0219621)
Supplement: S1 Table — (DOCX) [file pone.0219621.s001.docx]

**S1 Table.** Within-subject coefficients of variation (wCV) in ultrasound shear wave elasticity measurements according to target elasticity and acquisition depth

| **Variable** | **wCV (%)** | **95% confidence interval** |
| --- | --- | --- |
| **Overall** | 31.26 | 23.31–37.98 |
| **Target**^†^ |  |  |
| **8 ± 3 kPa** | 6.96 | 5.79–8.13 |
| **14 ± 4 kPa** | 7.69 | 6.15–9.38 |
| **25 ± 6 kPa** | 8.47 | 7.02–10.01 |
| **45 ± 8 kPa** | 10.43 | 8.81–12.15 |
| **80 ± 12 kPa** | 31.33 | 23.24–39.58 |
| **Depths including all targets**^††^ | | |
| **15 mm** | 13.06 | 9.56–16.15 |
| **30 mm** | 39.32 | 20.32–53.43 |
| **35 mm** | 18.24 | 13.75–22.91 |
| **60 mm** | 42.30 | 30.60–53.71 |
| **Depths including all targets except 80 ± 12 kPa**^††^ | | |
| **15 mm** | 5.09 | 3.82–6.24 |
| **30 mm** | 10.06 | 7.59–12.29 |
| **35 mm** | 11.56 | 8.19–14.49 |
| **60 mm** | 14.50 | 10.82–18.12 |

^†^The wCV was higher for targets with high (80 ± 12 kPa) rather than low elasticities (8 ± 3, 14 ± 4, 25 ± 6, and 45 ± 8 kPa ) (*p* < 0.001).

^††^ The wCV did not differ across acquisition depths.
