# Supplementary material for: Accuracy and precision of ultrasound shear wave elasticity measurements according to target elasticity and acquisition depth: A phantom study
Source: PLoS One. 2019 Jul 11;14(7):e0219621. doi: 10.1371/journal.pone.0219621 (PMC6622533; doi:10.1371/journal.pone.0219621)
Supplement: S2 Table — (DOCX) [file pone.0219621.s002.docx]

**S2 Table.** Shear wave elasticity measurements using five different elasticity targets at two different depths by a linear transducer

|  |  |  |  | Operator 1 |  | Operator 2 | |  | |
| --- | --- | --- | --- | --- | --- | --- | --- | --- | --- |
| Ultrasound  system | The size of ROI | Depths | 049 Phantom | Mean Elasticity (kPa) | Coefficient of variation (%) | | Mean Elasticity (kPa) | Coefficient of variation (%) |  |
| VTQ | 5 mm x 6 mm | 15mm | 8 ± 3 kPa | 7.5 | 0.5 | | 7.8 | 1.0 |  |
|  |  |  | 14 ± 4 kPa | 11.4 | 0.4 | | 12 | 1.4 |  |
|  |  |  | 25 ± 6 kPa | 18.8 | 0.7 | | 18.5 | 0.9 |  |
|  |  |  | 45 ± 8 kPa | 24.5 | 2.7 | | 25.4 | 2.3 |  |
|  |  |  | 80 ± 12 kPa | 8.4 | 8.5 | | 7.5 | 8.9 |  |
|  |  | 35mm | 8 ± 3 kPa | 7.2 | 0.6 | | 6.9 | 1.4 |  |
|  |  |  | 14 ± 4 kPa | 8.6 | 1.2 | | 9.1 | 0.5 |  |
|  |  |  | 25 ± 6 kPa | 16.6 | 0.9 | | 17.1 | 0.9 |  |
|  |  |  | 45 ± 8 kPa | 27.8 | 0.7 | | 28.3 | 1.0 |  |
|  |  |  | 80 ± 12 kPa | 13.1 | 58.9 | | 17.1 | 21.2 |  |
| VTIQ | 1.5 mm x 1.5 mm | 15mm | 8 ± 3 kPa | 10.7 | 4.1 | | 10.7 | 1.3 |  |
|  |  |  | 14 ± 4 kPa | 16 | 1.0 | | 15.6 | 1.8 |  |
|  |  |  | 25 ± 6 kPa | 22.4 | 1.0 | | 23.8 | 1.6 |  |
|  |  |  | 45 ± 8 kPa | 39.4 | 2.0 | | 37.4 | 2.2 |  |
|  |  |  | 80 ± 12 kPa | 56.5 | 2.6 | | 72.6 | 3.5 |  |
|  |  | 35mm | 8 ± 3 kPa | 10.5 | 2.1 | | 11 | 4.7 |  |
|  |  |  | 14 ± 4 kPa | 14.1 | 4.6 | | 13.4 | 3.1 |  |
|  |  |  | 25 ± 6 kPa | 22.1 | 4.0 | | 22.7 | 5.9 |  |
|  |  |  | 45 ± 8 kPa | 36.2 | 3.5 | | 35 | 2.7 |  |
|  |  |  | 80 ± 12 kPa | 51.0 | 4.9 | | 53.5 | 4.4 |  |
| Aixplorer | 10 mm in diameter | 15mm | 8 ± 3 kPa | 8.6 | 3.2 | | 8.6 | 4.9 |  |
|  |  |  | 14 ± 4 kPa | 11.2 | 1.2 | | 11.9 | 1.8 |  |
|  |  |  | 25 ± 6 kPa | 18 | 0.6 | | 18.3 | 0.8 |  |
|  |  |  | 45 ± 8 kPa | 25.2 | 1.6 | | 26.3 | 1.0 |  |
|  |  |  | 80 ± 12 kPa | 47.9 | 2.5 | | 47.6 | 3.8 |  |
|  |  | 35mm | 8 ± 3 kPa | 8.1 | 1.5 | | 8.3 | 1.4 |  |
|  |  |  | 14 ± 4 kPa | 9.5 | 1.5 | | 9.8 | 1.7 |  |
|  |  |  | 25 ± 6 kPa | 19.1 | 0.9 | | 19.5 | 0.8 |  |
|  |  |  | 45 ± 8 kPa | 35.7 | 2.9 | | 32.6 | 1.9 |  |
|  |  |  | 80 ± 12 kPa | 62.9 | 2.2 | | 62.1 | 1.8 |  |
| Aplio 500 | 9 mm in diameter | 15mm | 8 ± 3 kPa | 8 | 1.0 | | 7.8 | 1.3 |  |
|  |  |  | 14 ± 4 kPa | 11.9 | 0.7 | | 12 | 2.3 |  |
|  |  |  | 25 ± 6 kPa | 18.4 | 2.1 | | 18.8 | 1.6 |  |
|  |  |  | 45 ± 8 kPa | 23.9 | 3.0 | | 24.7 | 2.5 |  |
|  |  |  | 80 ± 12 kPa | 55.2 | 5.7 | | 54.6 | 4.3 |  |
|  |  | 35mm | 8 ± 3 kPa | 8.6 | 3.4 | | 8.2 | 8.3 |  |
|  |  |  | 14 ± 4 kPa | 11.2 | 5.8 | | 9.8 | 11.2 |  |
|  |  |  | 25 ± 6 kPa | 18.8 | 15.1 | | 16.5 | 6.4 |  |
|  |  |  | 45 ± 8 kPa | 31.8 | 1.0 | | 38.5 | 13.6 |  |
|  |  |  | 80 ± 12 kPa | 73.4 | 13.0 | | 87.7 | 13.2 |  |

ROI: region of interest
