# Supplementary material for: Accuracy and precision of ultrasound shear wave elasticity measurements according to target elasticity and acquisition depth: A phantom study
Source: PLoS One. 2019 Jul 11;14(7):e0219621. doi: 10.1371/journal.pone.0219621 (PMC6622533; doi:10.1371/journal.pone.0219621)
Supplement: S3 Table — (DOCX) [file pone.0219621.s003.docx]

**S3 Table.** Shear wave elasticity measurements using five different elasticity targets at two different depths by a curved transducer

|  |  |  |  | Operator 1 |  | Operator 2 |  |
| --- | --- | --- | --- | --- | --- | --- | --- |
| Ultrasound  system | The size of ROI | Depths | 049A Phantom | Mean  elasticity  (kPa) | Coefficient  of variation | Mean  elasticity  (kPa) | Coefficient of variation |
| EPIQ 5 | 10 mm x 12 mm | 30mm | 8 ± 3 kPa | 10.3 | 16.6 | 12.1 | 10.1 |
|  |  |  | 14 ± 4 kPa | 11.3 | 10.4 | 13.2 | 9.6 |
|  |  |  | 25 ± 6 kPa | 23.8 | 15.8 | 23.9 | 8.2 |
|  |  |  | 45 ± 8 kPa | 33.3 | 14.5 | 29.7 | 11.0 |
|  |  |  | 80 ± 12 kPa | 95.3 | 41.8 | 143.4 | 14.5 |
|  |  | 60mm | 8 ± 3 kPa | 10.9 | 8.9 | 10.5 | 8.8 |
|  |  |  | 14 ± 4 kPa | 11.2 | 7.7 | 13.1 | 5.1 |
|  |  |  | 25 ± 6 kPa | 22.0 | 11.5 | 25.7 | 5.7 |
|  |  |  | 45 ± 8 kPa | 28.1 | 9.4 | 26.3 | 24.0 |
|  |  |  | 80 ± 12 kPa | 15.8 | 70.8 | 65.9 | 14.5 |
| Aixplorer | 10 mm in diameter | 30mm | 8 ± 3 kPa | 9.4 | 1.8 | 9.3 | 1.1 |
|  |  |  | 14 ± 4 kPa | 9.6 | 1.7 | 9.6 | 1.2 |
|  |  |  | 25 ± 6 kPa | 21.4 | 2.8 | 22.3 | 2.8 |
|  |  |  | 45 ± 8 kPa | 31.3 | 2.0 | 30.9 | 2.7 |
|  |  |  | 80 ± 12 kPa | 49.5 | 2.7 | 48.3 | 3.1 |
|  |  | 60mm | 8 ± 3 kPa | 9.8 | 7.9 | 9.5 | 5.4 |
|  |  |  | 14 ± 4 kPa | 11.5 | 4.7 | 11.3 | 6.2 |
|  |  |  | 25 ± 6 kPa | 19.8 | 4.4 | 19.4 | 3.7 |
|  |  |  | 45 ± 8 kPa | 33.2 | 7.0 | 30.5 | 6.2 |
|  |  |  | 80 ± 12 kPa | 39.7 | 4.2 | 41.6 | 11.1 |
| Aplio 500 | 9 mm in diameter | 30mm | 8 ± 3 kPa | 8.7 | 3.1 | 8.9 | 2.8 |
|  |  |  | 14 ± 4 kPa | 9.8 | 1.6 | 10 | 3.6 |
|  |  |  | 25 ± 6 kPa | 20.6 | 1.3 | 20.2 | 4.3 |
|  |  |  | 45 ± 8 kPa | 33.8 | 6.0 | 37.7 | 5.6 |
|  |  |  | 80 ± 12 kPa | 80.5 | 3.6 | 84.6 | 6.0 |
|  |  | 60mm | 8 ± 3 kPa | 7.7 | 2.3 | 7.8 | 10.3 |
|  |  |  | 14 ± 4 kPa | 9.9 | 4.0 | 10 | 7.4 |
|  |  |  | 25 ± 6 kPa | 17.6 | 6.4 | 19.3 | 11.1 |
|  |  |  | 45 ± 8 kPa | 39.2 | 8.3 | 33.2 | 10.0 |
|  |  |  | 80 ± 12 kPa | 62.0 | 8.8 | 54.2 | 14.9 |

ROI: region of interest
