# Supplementary material for: Accuracy and precision of ultrasound shear wave elasticity measurements according to target elasticity and acquisition depth: A phantom study
Source: PLoS One. 2019 Jul 11;14(7):e0219621. doi: 10.1371/journal.pone.0219621 (PMC6622533; doi:10.1371/journal.pone.0219621)
Supplement: S4 Table — (DOCX) [file pone.0219621.s004.docx]

**S4 Table.** Measurement errors derived from differences between measured values and the margins of the target elasticity values, proportions of outliers, and within-subject coefficients of variation (wCV) according to transducers

|  | **Measurement errors (kPa)**^†^ | **Proportions of outliers (%)** | **wCV (%)**^††^ |
| --- | --- | --- | --- |
| **Linear transducer** |  |  |  |
| Overall | 5.86 | 51 (41 of 80) | 16.13 |
| 8 ± 3 kPa | 0.05 | 0 (0 of 16) | 5.22 |
| 14 ± 4 kPa | 0.22 | 31 (5 of 16) | 5.54 |
| 25 ± 6 kPa | 0.74 | 63 (10 of 16) | 7.17 |
| 45 ± 8 kPa | 6.64 | 81 (13 of 16) | 8.94 |
| 80 ± 12 kPa | 21.65 | 81 (13 of 16) | 16.43 |
| **Curved transducer** |  |  |  |
| Overall | 5.16 | 43 (26 of 60) | 40.82 |
| 8 ± 3 kPa | 0.17 | 8 (1 of 12) | 8.45 |
| 14 ± 4 kPa | 0.14 | 33 (4 of 12) | 10.14 |
| 25 ± 6 kPa | 0.20 | 8 (1 of 12) | 9.68 |
| 45 ± 8 kPa | 5.13 | 83 (10 of 12) | 12.00 |
| 80 ± 12 kPa | 20.15 | 83 (10 of 12) | 38.39 |

^†^Mean measurement errors and were higher for targets with high (45 ± 8 kPa and 80 ± 12 kPa) rather than low (8 ± 3, 14 ± 4, and 25 ± 6 kPa) elasticities (*p* < 0.016).

^††^The wCV were higher for targets with high (80 ± 12 kPa) rather than low (8 ± 3, 14 ± 4, 25 ± 6, and 45 ± 8 kPa) elasticities (*p* < 0.001).
