# Supplementary material for: Accuracy and precision of ultrasound shear wave elasticity measurements according to target elasticity and acquisition depth: A phantom study
Source: PLoS One. 2019 Jul 11;14(7):e0219621. doi: 10.1371/journal.pone.0219621 (PMC6622533; doi:10.1371/journal.pone.0219621)
Supplement: S5 Table — (DOCX) [file pone.0219621.s005.docx]

**S5 Table.** Measurement errors derived from differences between measured values and the margins of the target elasticity values, proportions of outliers, and within-subject coefficients of variation (wCVs) according to target elasticity in small targets

|  | **Measurement errors (kPa)**^†^ | **Proportions of outliers (%)** | **wCV (%)**^††^ |
| --- | --- | --- | --- |
| **10.4 mm** |  |  |  |
| Overall | 9.52 | 60 (24 of 40) | 22.30 |
| 8 ± 3 kPa | 0.31 | 25 (2 of 8) | 10.91 |
| 14 ± 4 kPa | 0.04 | 0 (0 of 8) | 7.86 |
| 25 ± 6 kPa | 1.41 | 75 (6 of 8) | 9.34 |
| 45 ± 8 kPa | 13.12 | 100 (8 of 8) | 11.29 |
| 80 ± 12 kPa | 32.71 | 100 (8 of 8) | 26.27 |
| **6.5 mm** |  |  |  |
| Overall | 12.37 | 65 (26 of 40) | 14.32 |
| 8 ± 3 kPa | 2.69 | 100 (8 of 8) | 8.13 |
| 14 ± 4 kPa | 0.00 | 0 (0 of 8) | 6.55 |
| 25 ± 6 kPa | 1.22 | 75 (6 of 8) | 10.84 |
| 45 ± 8 kPa | 16.2 | 100 (8 of 8) | 10.01 |
| 80 ± 12 kPa | 41.73 | 100 (8 of 8) | 19.06 |

^†^Mean measurement errors were higher for targets with high (45 ± 8 kPa and 80 ± 12 kPa) rather than low (8 ± 3, 14 ± 4, and 25 ± 6 kPa) elasticities (*p* < 0.016).

^††^The wCV were higher for targets with high (80 ± 12 kPa) rather than low (8 ± 3, 14 ± 4, 25 ± 6, and 45 ± 8 kPa) elasticities (*p* < 0.001).
