# Supplementary figures and images for: Accuracy and precision of ultrasound shear wave elasticity measurements according to target elasticity and acquisition depth: A phantom study
Source: PLoS One. 2019 Jul 11;14(7):e0219621. doi: 10.1371/journal.pone.0219621 (PMC6622533; doi:10.1371/journal.pone.0219621)

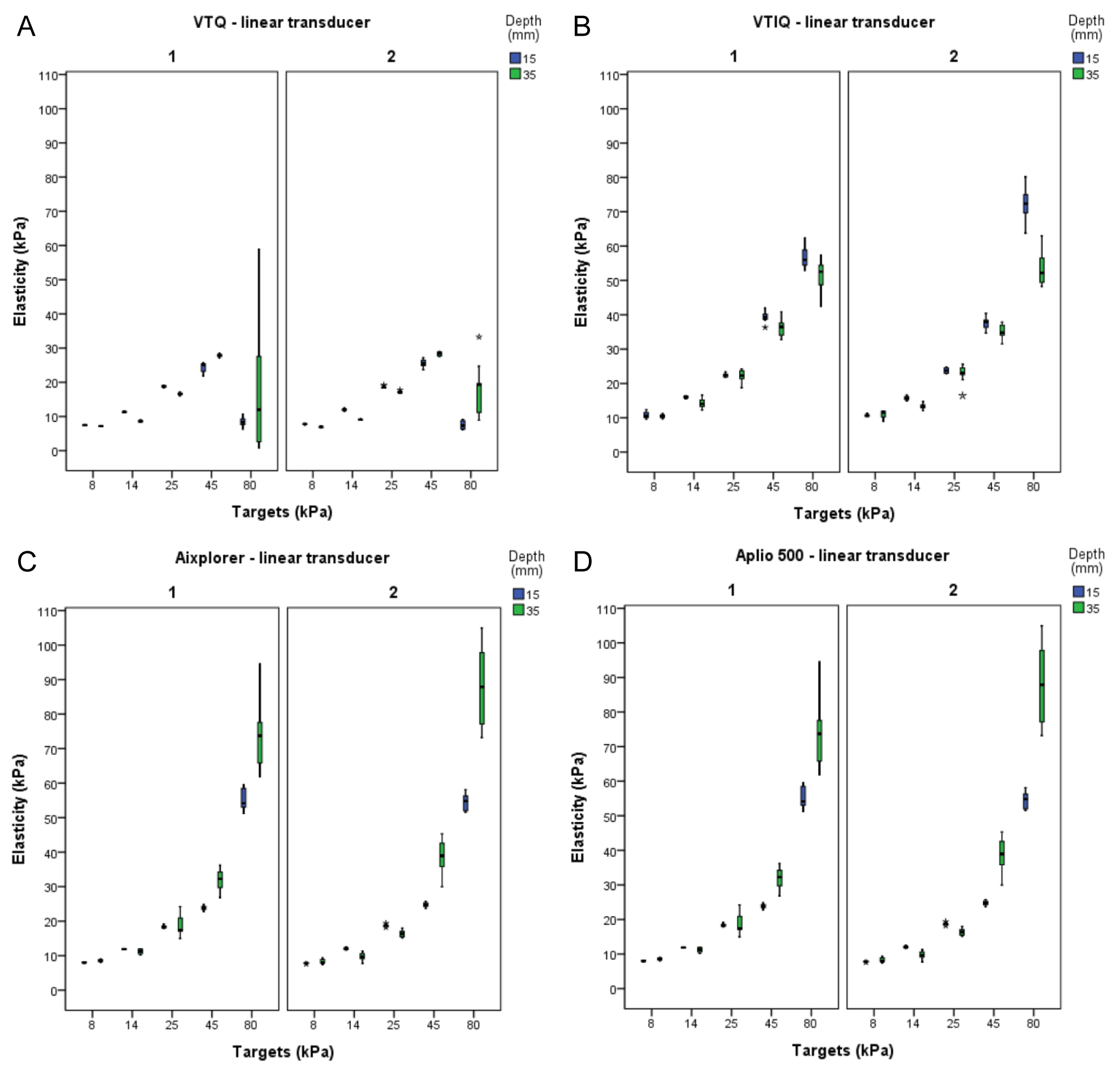

Supplement: S1 Fig — A. a linear transducer of VTQ, B. a linear transducer of VTIQ, C. a linear transducer of Aixplorer, and D. a linear transducer of Aplio 500. (TIF) [file pone.0219621.s006.tif]

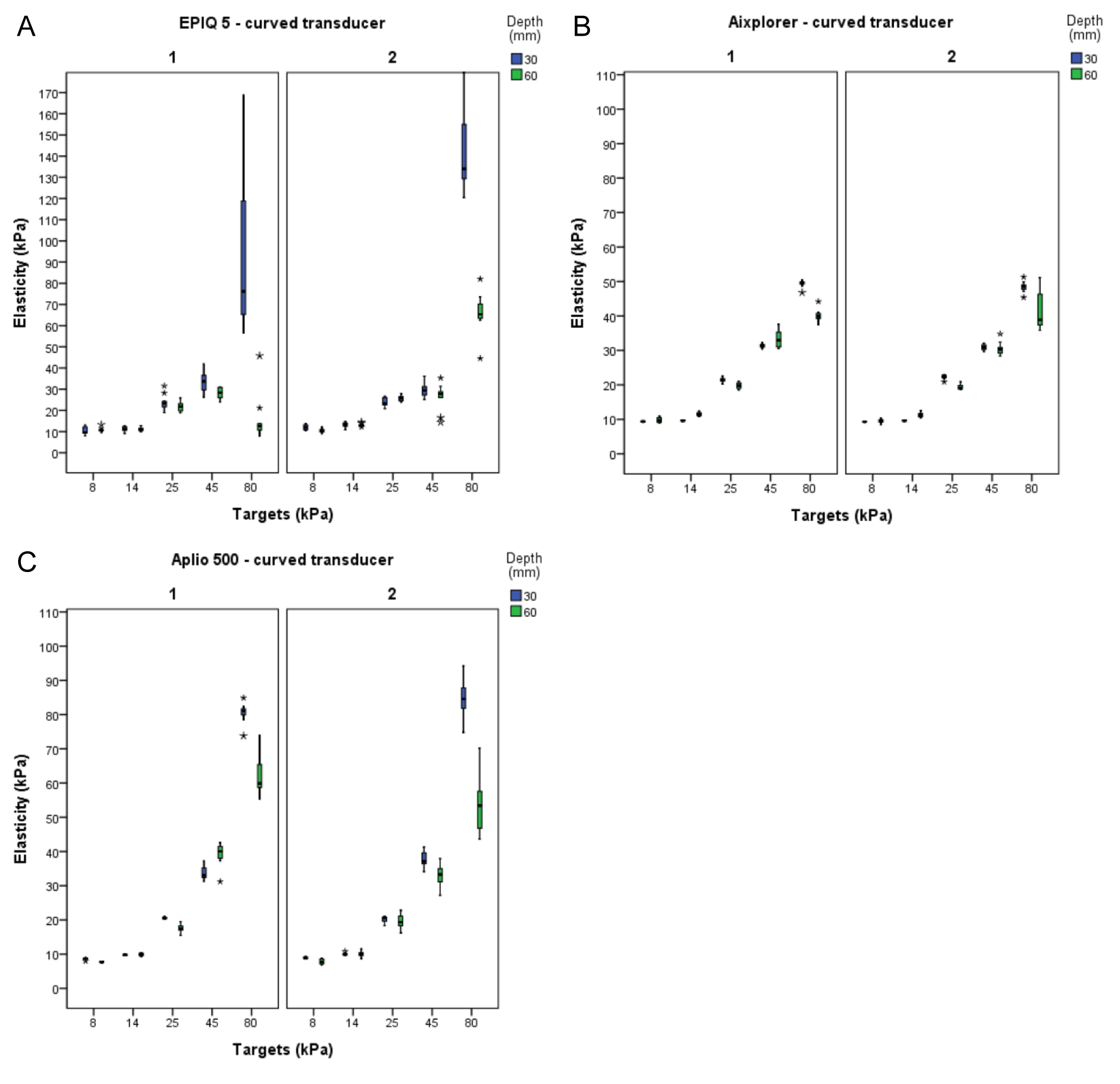

Supplement: S2 Fig — A. a curved transducer of EPIQ 5, B. a curved transducer of Aixplorer, and C. a curved transducer of Aplio 500. (TIF) [file pone.0219621.s007.tif]
